# Single-Kernel Ionomic Profiles Are Highly Heritable Indicators of Genetic and Environmental Influences on Elemental Accumulation in Maize Grain (*Zea mays*)

**DOI:** 10.1371/journal.pone.0087628

**Published:** 2014-01-29

**Authors:** Ivan R. Baxter, Gregory Ziegler, Brett Lahner, Michael V. Mickelbart, Rachel Foley, John Danku, Paul Armstrong, David E. Salt, Owen A. Hoekenga

**Affiliations:** 1 United States Department of Agriculture, Agricultural Research Service, Plant Genetics Research Unit, Donald Danforth Plant Science Center, St. Louis, Missouri, United States of America; 2 Purdue University, Department of Horticulture and Landscape Architecture, West Lafayette, Indiana, United States of America; 3 University of Aberdeen, Institute of Biological and Environmental Science, Aberdeen, United Kingdom; 4 United States Department of Agriculture, Agricultural Research Service, Engineering and Wind Erosion Research Unit, Manhattan, Kansas, United States of America; 5 United States Department of Agriculture, Agricultural Research Service, RW Holley Center for Agriculture and Health, Ithaca, New York, United States of America; National Key Laboratory of Crop Genetic Improvement, China

## Abstract

The ionome, or elemental profile, of a maize kernel can be viewed in at least two distinct ways. First, the collection of elements within the kernel are food and feed for people and animals. Second, the ionome of the kernel represents a developmental end point that can summarize the life history of a plant, combining genetic programs and environmental interactions. We assert that single-kernel-based phenotyping of the ionome is an effective method of analysis, as it represents a reasonable compromise between precision, efficiency, and power. Here, we evaluate potential pitfalls of this sampling strategy using several field-grown maize sample sets. We demonstrate that there is enough genetically determined diversity in accumulation of many of the elements assayed to overcome potential artifacts. Further, we demonstrate that environmental signals are detectable through their influence on the kernel ionome. We conclude that using single kernels as the sampling unit is a valid approach for understanding genetic and environmental effects on the maize kernel ionome.

## Introduction

Increasing agricultural sustainability requires improvements in nutrient use efficiency while decreasing fertilizer inputs. These requirements exist as the majority of arable soils have limitations associated with them [1]. Only 16% of crop lands are “without constraint,” and most of these constraints are related to elements found in inadequate or excessive amounts [1]. The range of soil elemental concentrations optimal for productive growth of crops is much smaller than that of the wild plants they may have displaced. This is likely due to human selection of crop plants for yield under optimal agricultural conditions and not for adaptive mineral nutrient efficiency on poor soil. Non-optimal concentrations of many elements limit the productivity of crops or necessitate significant inputs to maintain productivity. Major elemental limitations include excessive Na [2]; insufficient N [3], P [4], and K [5]; acid soil syndrome, which causes Al, Mn, and Fe toxicity and Mo, Ca, and P deficiency [6]; and Fe deficiency in alkaline soils [7]. Due to low soil fertility and the effects of poverty (e.g., inability to buy fertilizer), crop yields in most of Africa are less than one-fifth of U.S. yields [8]. In order to meet future food needs, we will need to increase yields while increasing the sustainability of agricultural systems in both developed and developing countries. In order to develop crops that can grow in diverse soils with less fertilizer, we require a deeper understanding of the genes that allow plants to adapt to different soil environments [9].

The elemental composition of a cell, tissue, or organism is referred to as the ionome [10]. The ionome can be profiled using high-throughput, high-accuracy analytical chemistry such as inductively coupled plasma-mass spectrometry (ICP-MS), which can measure the concentrations of 20 elements over 5 logs in ∼2 minutes per sample. To apply this systems biology phenotyping platform most efficiently, the best tissue for estimating the ionome of a crop plant must be used [9], [11], [12]. We assert that mature seeds are the ideal tissue when resources are limited, as mature seeds represent a well-defined developmental end point that summarizes the life history and genetic composition of a particular individual. Seeds are also highly stable and are easy to store, transport, and handle. Furthermore, seeds are feedstocks for people, animals, and industrial processes such that the seed ionome alone is of high value and represents an excellent proxy for a whole plant.

In an ideal world, a survey of tissues could be used to track the ionome through developmental time. However, genetic and environmental determinants make this approach difficult to implement on large populations of field-grown plants, as diverse varieties may progress through development at different rates that may be more or less influenced by daily weather or other environmental factors. Compromises are required to ensure the success of a particular research program, especially one that aims to identify genes that are effective over a range of environments rather than emphasizing a single one. We propose that the analysis of single seeds is the most efficient use of resources to characterize a highly relevant ionome for field-grown crops. Intact seeds are at reduced risk for contamination or preparative artifacts due to sample grinding and are an easily automated sample unit, and the overall reduced cost of preparation and analysis make this the best compromise of efficiency, relevance, and precision. This scheme is not without obvious potential problems, however, not the least of which is heterogeneity between seeds produced by the same plant or related plants within an experimental plot that may shape the estimation of the ionome through single-seed-based observation.

In the present study, we test the premise of confounding heterogeneity to better understand the sources of variance that contribute to the seed ionome using maize kernels. We assert that single-seed-based analysis is a reasonable strategy for phenotypic analysis, especially when resources are limited and considerations of the number of test environments are balanced against accuracy within any single environment. The ionomic profiling workflow described in this study for maize kernels takes advantage of automation for sample handling, weighing, and liquid dispensing to reduce operator time, effort, and overall cost. This optimization allows 576 kernels to be analyzed from start to finish in 3 days. We demonstrate the utility of our workflow as an effective means of collecting ionomic data relevant to increasing agricultural sustainability.

## Results

### Experimental Design and Analysis

The ionomics pipeline starts with arraying the single kernels in 48 well plates that are then loaded onto the custom-built weighing robot. Each kernel is weighed and deposited into a glass digestion tube. The samples are digested down to the elemental components using heat and acid. The digested material is analyzed by ICP-MS to quantitatively measure the concentrations of 20 elements with high precision. Both internal and external standards are used to correct for instrument drift during and between experiments.

**Figure 1:**
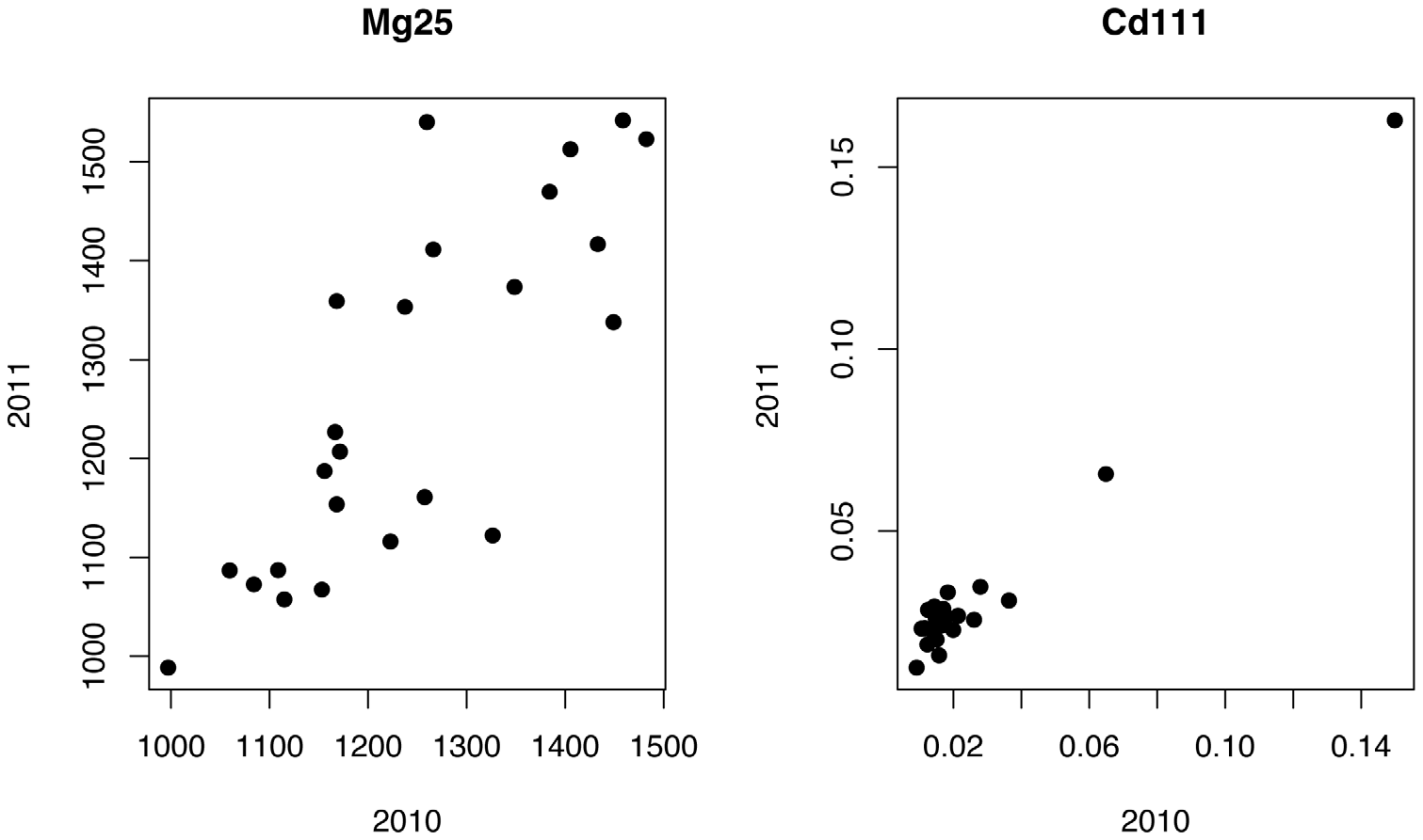
Correlation between 23 NAM parents across two years. The plot means of all 5 or 6 replicate plots for each NAM parent line (excluding Il14H, HP301 and P39) were averaged for each year.

The 26 parents of the maize nested association mapping (NAM) panel (hereafter, the NAM Founders) [13] were grown in a randomized complete block design, in 2010 (six blocks) and 2011 (five blocks), at the Purdue Agronomy Center for Research Education. Kernels from the middle of 1–2 cobs per plot were removed from the cob by hand and analyzed using the ionomics pipeline. For each plot (i.e., each genotype within a block), four to six seeds were analyzed. Outliers within each plot were removed using a conservative cutoff of 15 median absolute deviations from the plot median (derived from [14]) on an element by element basis. To be even more conservative, we used the mean of all the samples in each plot and omitted the one popcorn and two sweet corn entries in the NAM Founders in order to prevent artifacts from low seed weights from biasing the analysis. To analyze the sources of variance, a simple linear model with line and year effects was used to demonstrate that all 20 elements had a significant effect of genotype (p<0.01 with a Bonferroni correction; Table 1). Most elements also had significant effects of year as well as line x year interactions. There were four potentially problematic elements characterized by low heritability and or low correlation between seasons: B, Na, Al, and Se. This result was not unexpected, as all are in low abundance in the kernel and have several analytical interferences (see discussion). Narrow sense heritability estimates within a single season were quite high, with 17 and 19 elements having heritabilities >0.5 in each year respectively. Heritability estimates decreased when both years were considered in a single model, likely due to the significant line x year interactions. Twelve elements had a two-year heritability estimate above 0.5. For 16 elements there was a statistically significant (p<0.01) correlation between the two years (see examples in Figure 1).

**Table 1:** Analysis of the NAM founders ionomic profiles from 2010 and 2011.

	Geno	Year	GxY	h2 2010	h2 2011	h2 both	Corr	JK 5% p val	JK 5% h2
Weight	1.3E-31	2.6E-04	1.2E-05	0.64	0.67	0.53	0.65	9.9E-19	0.50
B	3.3E-23	6.6E-17	2.5E-09	0.63	0.56	0.37	NS	6.1E-06	0.30
Na	1.5E-14	9.4E-75	NS	0.28	0.52	0.12	0.76	3.3E-03	0.11
Mg	1.2E-62	NS	5.3E-11	0.81	0.84	0.73	0.80	1.6E-33	0.61
Al	4.2E-09	2.2E-57	4.2E-04	0.3	0.43	0.12	NS	1.5E-02	0.10
P	2.0E-49	6.7E-69	2.7E-07	0.81	0.71	0.38	0.77	1.5E-25	0.39
S	3.7E-40	1.3E-07	8.1E-13	0.81	0.61	0.55	0.55	2.2E-25	0.52
K	4.4E-27	NS	3.8E-11	0.65	0.65	0.46	NS	3.9E-14	0.44
Ca	2.6E-30	7.2E-06	8.5E-08	0.6	0.69	0.5	0.59	9.3E-15	0.34
Mn	2.0E-44	1.8E-22	8.7E-11	0.78	0.71	0.54	0.64	5.6E-33	0.60
Fe	4.0E-51	NS	NS	0.74	0.8	0.7	0.85	1.3E-31	0.21
Co	3.6E-22	NS	NS	0.5	0.54	0.5	0.95	4.1E-17	0.36
Ni	1.8E-24	1.2E-21	2.1E-04	0.6	0.58	0.39	0.74	4.1E-22	0.38
Cu	7.6E-71	6.9E-12	2.5E-06	0.84	0.86	0.77	0.88	3.4E-49	0.66
Zn	3.4E-61	5.2E-23	9.8E-08	0.81	0.83	0.67	0.81	1.0E-46	0.63
As	5.3E-30	NS	4.2E-05	0.66	0.62	0.52	0.64	3.4E-16	0.52
Se	2.7E-08	3.6E-04	NS	0.47	0.28	0.25	NS	3.7E-03	0.27
Rb	2.6E-23	1.4E-36	NS	0.54	0.64	0.32	0.77	1.1E-19	0.30
Sr	4.7E-21	1.9E-31	2.1E-04	0.53	0.57	0.31	0.62	1.2E-08	0.23
Mo	2.5E-29	NS	NS	0.6	0.63	0.55	0.86	4.6E-25	0.51
Cd	5.0E-63	7.5E-05	NS	0.75	0.9	0.79	0.98	4.1E-37	0.32

Geno: p value for the genotype term in an ANOVA of a linear model with genotype and year and their interaction. Year: p value for the year term. GxY: p value for the genotype by year interaction. The significance cutoff for the first three columns was set at p<0.0005 to account for the multiple testing correction. NS: not significant. h2: the narrow sense heritability for each year and combined. Corr: the correlation between the line average from 2010 and 2011. The last two rows are the results from the jackknife analysis of 100 datasets with a single seed per plot. JK 5% p val: the 5% most significant p value for the genotype term in the two year model (i.e., 95% of the time the p value was more significant). JK 5% h2: the 5% highest heritability across two years.

In these ionomic studies, we wished to emphasize the potential to detect QTLs in plants grown across a larger number of different environments. This requires a compromise between population size, number of field locations, and sampling depth for any one accession. To estimate if a single kernel per cob would be a feasible approach to analyzing large populations across multiple environments, we created 100 datasets by randomly sampling one of the four to six analyzed samples from each plot, a process known as jackknifing. Since the outlier removal that we performed before the first analysis was based on the distribution of samples in a given plot, we used the original (without correction for outliers) dataset for this simulation. The distribution of jackknifed heritabilities at the 5% value over all data (i.e., 95% of the single-seed dataset heritabilities were greater than these values) was lower than those calculated for means, but 7 elements had heritability >0.45 and 11 were >0.35 (Table 1). We found this a reasonable compromise, in order to detect potential QTLs from a larger number of environments at different locations rather than focusing all of our attention on a single location.

### Potential Confounding effects

To evaluate potential confounding effects that would limit our ability to detect genetic, environment, or gene by environment effects, we examined several potential sources of variance.

#### Outcrossing

Open pollinated ears are likely to have some kernels fertilized by pollen from nearby rows. If the paternal genotype has a significant effect on the kernel ionome, this genetic contamination may confound the genetic signal from the maternal plant. While we did not specifically test for this effect in these experiments, the random block design of the NAM parent experiment should have randomized the genotypes of the contaminating pollen and therefore contributed to the unexplained variance detected in that experiment. The heritabilities observed in that experiment (Table 1) suggest that this potential contamination will not prevent the detection of genetic effects, at least at the plot spacing used in these experiments (76 cm).

#### Cob location

**Figure 2:**
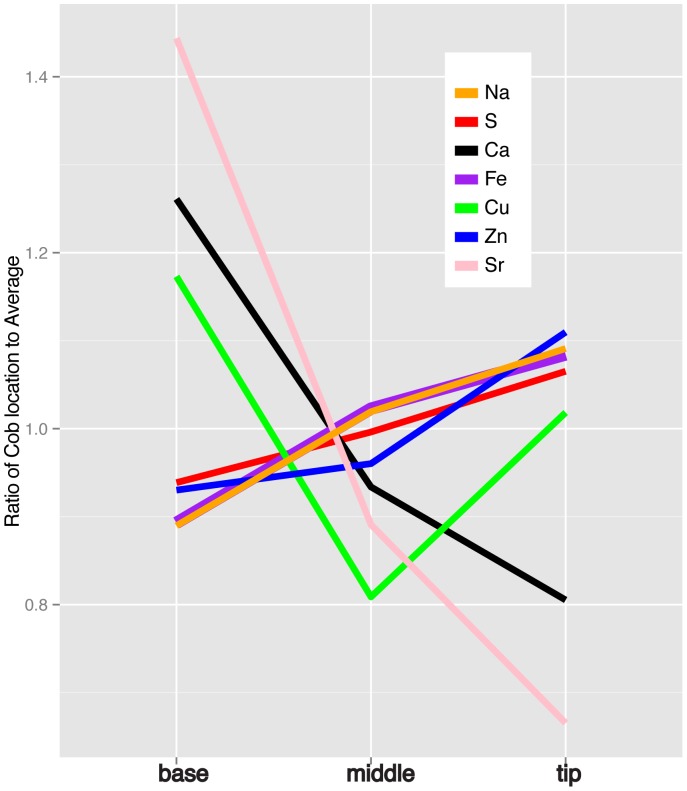
Gradients in elemental accumulation along the cob. For each ear, the samples for each section were averaged and then normalized to the mean of the ear. The values for all ears were then averaged. Only elements with significant effects of cob location were included in the model.

Given the differences in seed filling along the ear, elemental accumulation gradients could exist from the base to the tip of the ear. To determine the prevalence and magnitude of these gradients, we analyzed eight kernels from the base, middle, and tip of three independent ears from plants grown at four different agricultural research stations in Missouri, Texas, and Iowa. ANOVA indicated a significant effect (p<0.01 with Bonferroni correction) of position within the cob for seven elements (Table 2 and Figure 2). However, the location of the experimental fields accounted for much more of the variance in all elements except Na, S, Ca, and Zn. Additionally, the dynamic range of elemental accumulation (defined by the ratio of maximum accumulation for a cob location to minimum accumulation for a cob location) was well below the dynamic range of the observations from the two years of NAM Founders in Indiana. For example, while the highest and lowest cob locations differed by an average of 49% for Cu, the highest Cu accumulating line was over 500% higher than the lowest line in both 2010 and 2011. These data suggest that the variation due to cob location will not prevent identification of genetic environmental effects for most elements.

**Table 2:** Analysis of the seed position on the cob on B73 ionomic profiles.

	Dyn Range Cob	Dyn Range 2010	Dyn Range 2011	Farm p val	Field p val	Cob p val	Farm SSperc	Field SSperc	Cob SSperc
B	1.40	1.81	1.69	NS	NS	NS	0.05	0.06	0.31
Na	1.32	2.59	4.00	NS	NS	4.3E-04	0.19	0.04	0.33
Mg	1.08	1.51	1.59	3.2E-11	NS	NS	0.83	0.00	0.01
Al	1.50	2.00	3.00	2.0E-04	NS	NS	0.40	0.00	0.20
P	1.08	1.52	1.43	7.3E-14	NS	NS	0.85	0.01	0.05
S	1.15	1.64	1.56	6.9E-05	NS	3.6E-05	0.36	0.00	0.33
K	1.08	1.80	1.77	5.8E-06	NS	NS	0.54	0.05	0.07
Ca	1.60	3.50	4.07	2.1E-08	NS	1.4E-08	0.44	0.01	0.40
Mn	1.22	2.70	3.63	3.9E-05	NS	NS	0.46	0.02	0.15
Fe	1.24	2.11	2.11	2.6E-10	NS	7.9E-07	0.61	0.01	0.24
Co	1.55	7.84	11.02	NS	NS	NS	0.31	0.04	0.06
Ni	1.26	7.33	9.72	9.7E-16	NS	NS	0.88	0.03	0.02
Cu	1.49	5.78	5.45	2.2E-09	NS	5.7E-07	0.55	0.02	0.28
Zn	1.24	1.98	2.62	NS	NS	3.6E-04	0.18	0.02	0.35
As	1.58	2.65	3.71	8.2E-12	NS	NS	0.84	0.01	0.00
Rb	1.16	2.41	2.77	6.3E-18	NS	NS	0.92	0.01	0.02
Sr	2.30	3.71	3.94	5.8E-11	NS	4.9E-07	0.64	0.00	0.23
Mo	1.21	6.50	3.64	1.5E-21	NS	NS	0.97	0.00	0.00
Cd	1.73	16.30	13.01	NS	NS	NS	0.43	0.00	0.01

Dyn Range Cob: the average dynamic range of values (max accumulation of a position on a single cob/min accumulation on the same cob) from different positions on the cob. Dyn Range 2010 and 2011: the dynamic range in genotype values within that year (max accumulating genotype/min accumulating genotype). Farm p val: p value for the farm term in an ANOVA of a linear model with farm, field location, and location on cob. Field p val: p value for the field location term. Cob p val: p value for the location on cob term. The significance cutoff was set at p<0.0005 to account for the multiple testing correction. NS: not significant. Farm, Field and Cob SSperc are the percentage of the variance (sum of squares) accounted for by the various terms. Selenium was not measured in this experiment.

#### Seed composition

**Figure 3:**
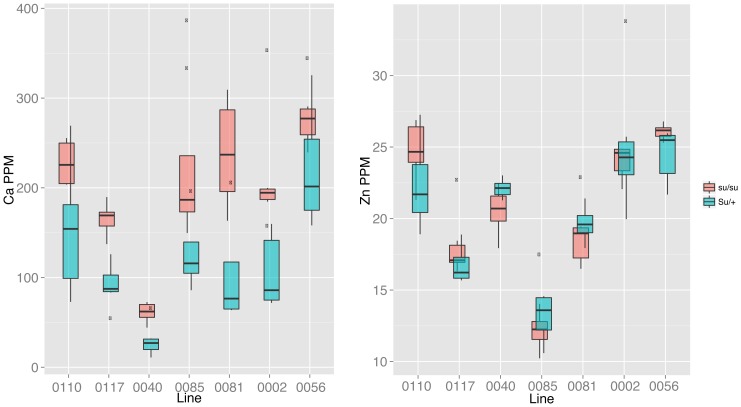
Alphabox plots of the effect of *su* on Ca and Zn accumulation. Five number summaries (median, 1^st^ and 3^rd^ quartiles, and 1.5 interquartile range) are shown of each seed type. Salmon denotes the values for the *su/su* (collapsed) seed, while teal denotes the values for *Su*/+ (plump) alleles. Outliers beyond 1.5 IQR are shown as black dots. Line names are the entry numbers in the Panzea database for the B73 x IL14H (Z011) population.

Elements are not distributed uniformly throughout the seeds of grain crops: some elements are concentrated in the embryo (e.g., Fe and Zn), while others (Ca and S) have substantial accumulation in the maternal tissues of the pericarp and endosperm [15]–[18]. Large differences in amounts of these compartments, such as differences in the organic composition of the seed, could change the total elemental composition of the kernel. The *sugary* locus is a key step in starch biosynthesis, with mutations at *sugary* creating the sweet corn many people eat as a fresh vegetable. Furthermore, mutant kernels also have higher protein levels than wild-type siblings [19]. As *sugary* kernels mature and dehydrate, they shrink, losing more stored carbon than Su+ seeds, and can potentially lose water-soluble ions [20]. To determine the effect of *sugary* on the elemental composition, we identified seven RI lines from the dent x sweet, B73 x IL14H NAM subpopulation where *sugary* was still segregating. We separated the seeds from single plots based on the kernel morphology (collapsed *su/su* seeds from plump *Su*/+ seeds) and analyzed their elemental content (Table 3 and Figure 3). For nine of the elements, there was a significant effect of the *sugary* locus, and for all but Fe and As, a significant effect of line could also be detected. We also analyzed a single seed per entry from this population. With this limited sampling, we were able to identify 31 QTLs (Table 4), including 8 strong QTLs with LOD scores >5 (highest 95% permutation threshold for any element was 2.8). We identified QTLs for P, K, Ni, and Cu that localized with *sugary* on Chr 4. The presence of *sugary* did not, however, prevent us from identifying an additional 3, 2, and 1 QTLs for Cu, Ni, and P, respectively, demonstrating that the gross change in kernel composition did not obscure all other genetic signals.

**Table 3:** Analysis of the sugary status on ionomic profiles.

	Line	Su
weights	2.5E-15	4.02E-09
B	2.2E-05	NS
Na	1.3E-05	NS
Mg	NS	NS
Al	4.7E-05	NS
P	2.9E-12	3.9E-05
S	6.7E-17	1.3E-10
K	2.6E-13	1.2E-16
Ca	8.7E-14	2.3E-09
Mn	2.1E-27	8.6E-08
Fe	NS	1.2E-08
Co	NS	NS
Ni	1.6E-10	NS
Cu	3.3E-23	NS
Zn	2.9E-23	NS
As	NS	4.4E-06
Se	1.9E-06	5.8E-06
Rb	2.7E-32	5.4E-11
Mo	1.9E-27	NS
Cd	1.6E-24	NS

Line: p value for the genotype term in an ANOVA of a linear model with line and *sugary* genotype. Su: p value for the sugary term. The significance cutoff was set at p<0.0005 to account for the multiple testing correction. NS: not significant.

**Table 4:** QTLs identified in the B73 x IL14H population.

El	Chm	Pos (cM)	LOD	MI Start	MI Stop	R^2^	SumR^2^	Add.Eff	PT	Co	Nearest marker
B	3	123.5	3.3	121.9	129.8	0.08	0.13	−58.93	2.5	7	PZB01457.1
B	5	13.3	2.5	13.3	13.3	0.05	0.13	−51.74	2.5	7	PHM13122.43
Cd	2	84.2	17.4	63.0	102.8	0.38	0.38	−0.02	2.6	1	PZA00495.5
Co	1	193.1	2.9	190.0	193.1	0.06	0.15	0.00	2.4	10	PZA00235.9
Co	5	23.2	3.2	13.3	29.1	0.10	0.15	0.00	2.4	10	PZA01925.1
Cu	2	94.5	4.3	88.6	100.0	0.09	0.24	−0.22	2.8	10	PZA01735.1
Cu	4	56.6	3.1	56.0	59.9	0.06	0.24	−0.20	2.8	10	PZA00218.1
Cu	8	80.6	4.5	74.1	86.8	0.10	0.24	0.23	2.8	10	PZA03698.1
K	4	54.9	5.0	49.7	59.9	0.15	0.15	−478.96	2.7	7	PZA01751.2
Mn	1	34.3	4.1	33.9	35.7	0.06	0.57	−0.91	2.8	10	PZA01030.1
Mn	1	89.1	14.4	73.4	106.0	0.30	0.57	−1.45	2.8	10	PZA02135.2
Mn	3	91.8	5.1	83.6	97.9	0.10	0.57	0.91	2.8	10	PZA03735.1
Mn	5	7.2	3.4	7.2	10.3	0.05	0.57	0.61	2.8	10	PZA01438.1
Mn	5	40.8	3.6	34.7	49.2	0.06	0.57	−0.66	2.8	10	PZA01284.6
Mo	1	53.9	3.0	50.6	53.9	0.05	0.33	−0.01	2.8	10	PZA02686.1
Mo	1	147.4	14.6	129.8	155.8	0.29	0.33	−0.03	2.8	10	PZA02269.3.4
Na	5	13.3	3.0	10.3	21.2	0.06	0.14	−25.24	2.5	10	PHM13122.43
Na	5	43.7	3.7	36.8	49.2	0.08	0.14	29.13	2.5	10	PZA01327.1
Ni	1	149.9	8.7	136.2	163.7	0.15	0.35	0.06	2.7	10	PZA02269.3.4
Ni	2	91.7	3.4	70.5	100.0	0.06	0.35	−0.04	2.7	10	PZA01735.1
Ni	4	56.6	3.3	56.0	59.9	0.06	0.35	0.04	2.7	10	PZA00218.1
Ni	5	71.2	3.6	60.7	84.6	0.08	0.35	0.04	2.7	10	PZA03536.1
P	4	53.9	4.0	52.4	56.6	0.08	0.13	−191.73	2.7	10	PZA01751.2
P	6	33.4	3.0	33.4	35.1	0.05	0.13	−127.55	2.7	10	PZA03461.1
Rb	3	119.9	2.6	118.0	121.9	0.05	0.25	−0.23	2.4	10	PZA02516.1
Rb	4	127.1	2.6	127.1	127.1	0.05	0.25	0.24	2.4	10	PZA03322.5.3
Rb	5	101.2	3.0	101.2	104.5	0.05	0.25	−0.27	2.4	10	PZA02411.3
Rb	8	29.6	5.4	17.5	41.2	0.10	0.25	0.34	2.4	10	PZA03178.1
S	1	161.7	4.1	151.9	173.5	0.09	0.09	−117.13	2.5	9	PZA02698.3
Zn	5	99.3	5.5	91.7	109.5	0.12	0.21	−2.51	2.3	7	PZA02411.3
Zn	7	132.4	3.8	123.1	132.4	0.09	0.21	2.12	2.3	7	PZA01744.1

El: Element. Chm: Chromosome. Pos: Position of QTL in cM. LOD: LOD score. MI Start and Stop: the mapping intervals defining where the LOD trace crossed the 95% permutation threshold. R^2^: the fraction of variance accounted for by the QTL, Sum R^2^: The cumulative fraction of variance accounted for by all of the QTLs for the element. Add.eff: The additive effect of the QTL in PPM. PT: the 95% permutation threshold from 1000 permutations. CO: number of cofactors used in the model. Nearest Marker: The nearest marker.

## Discussion

A defined developmental stage that is stable at room temperature, the mature kernel has many inherent advantages as a sample tissue. Single kernels are discrete, relatively small samples that can be easily manipulated by technicians or robots. Single-kernel-based analyses also avoid mechanical disruption (e.g., grinding or milling) that are labor intensive and/or introduce the possibility of contamination from the tools used. With these experiments, we have demonstrated that single-seed measurements of elemental profiles can be used to study the genetic and environmental effects on seed elemental accumulation.

Developing the ionomics pipeline around single-kernel sampling has enabled the processing of 1728 samples per week at the USDA-ARS/Danforth Center profiling facility. Multiple kernels can be profiled with less cost and effort than would be required to pool, disrupt and homogenize, subsample, digest, and run as a single composite sample. This provides several advantages from an analytical and statistical perspective. With multiple measurements from a given packet, outliers resulting from the analytical process or machine error can be discarded, as we did here. The measurements can be averaged to a single value or included as nested factors in a statistical model.

We investigated three potential confounding effects: outcrossing, relative position of the seed within the ear, and the composition of the organic components of the seeds. While we were able to detect statistically significant effects for the latter two factors for subsets of elements, neither one of them was large enough to obscure the signal from genetic or environmental factors.

Since we did not explicitly test for outcrossing, instead conducting an experiment, where it could occur, that tested whether the ionomic traits were still heritable, we cannot estimate directly the magnitude of the effects in this study. However, the high heritabilities observed in these experiments suggest that the effects are small. Of the range of processes that can contribute to the seed ionome, root uptake, partitioning, shoot leaf transport, leaf partitioning, leaf remobilization, leaf seed transport, and seed loading are all exclusively maternal, while embryo uptake has contributions from both maternal and paternal alleles. When viewed from this perspective, it seems reasonable that paternal effects are a minor contribution to the ionome. However, we cannot exclude the possibility that there are loci that will have a strong paternal effect.

The high heritability observed in jackknifed datasets of a single seed per plot from the NAM Founders, without the benefit of outlier removal, demonstrates that the genetic variation for many elemental traits in maize is large enough that most confounding factors will not interfere with analysis. However, if the labor and resources are available, controlled pollination experiments and harvesting only the center of the cob will reduce the variability from pollination and cob location. Analysis of the organic composition of the kernels through NIR spectroscopy or other methods could allow for differences in protein, oil, and starch content to be included as cofactors in statistical models.

The appropriate number of seeds for analysis will depend on the experimental design and expected frequencies of functionally distinct alleles. For quantitative genetic experiments such as recombinant inbred populations with several hundred lines (or thousands. in the case of the NAM), where a given allele may occur over a hundred times, a single seed per line may suffice to identify significant QTLs. From a technical standpoint, more observations will decrease the associated error but be biologically unnecessary to find common alleles, and may reduce the number of lines examined if you have fixed capacity for analysis. In those cases where rare alleles have large phenotypic effects, such as in an association mapping population or when a new mutation is being tested, more seeds will be required to increase the confidence of detection [21]. While we were able to identify significant QTLs with a single seed in the B73 x IL14H population, our standard practice when analyzing a single RIL population is to profile two to four seeds per plot. When analyzing extremely large populations such as the Maize NAM, which has >6000 plots in the standard experimental design, profiling one or two seeds from most lines allows for the analysis of the population in more locations [13], [22]. This compromise in technical accuracy of the ionomic phenotype versus increasing the number of environments is a highly reasonable one to make, given that resources are almost always limiting.

There are, of course several drawbacks and limitations to the approach we have taken. By opting for high throughput while analyzing 20 elements simultaneously, we do lose some sensitivity compared to lower throughput efforts focused on fewer elements. Examples of this include using standard glass (sodium borosilicate) tubes for chemical digestion, which increases the background levels of Na and B; not using a dynamic reaction cell on the ICP-MS, which leads to isobaric interferences for Se, As, and Al; and the use of a single sample dilution factor to measure elements with concentrations ranging from several hundred parts per million to low parts per billion.

This speed/number of elements/accuracy trade-off, combined with the low abundance of some of the elements in maize kernels, leads to several elements being at or below the level of detection in many or most of the samples analyzed. We have considered omitting these samples from the analysis pipeline entirely, but currently include them for occasions where a given mutant line or diverse accession accumulates sufficient qualities to exceed the detection threshold, such as for two of the NAM parents for Cd (Figure 1).

The single-seed analysis approach has the potential to create two major artifacts. One is that differences in weights can cause perceived differences in accumulation in elements at or below detection limits when the signal from the instrument is normalized to weight. The second is that some elements may be restricted to a specific subcompartment of the seed, such as the embryo, so alterations in the other compartments may not affect the amount of the element, even though they would affect the weight. However, single-seed analysis, with the weight recorded and included in every dataset, allows for the dataset to be analyzed from an amount per seed perspective, which would alleviate these issues (although it may create artifacts in itself). We have opted to present the data as a concentration in the seed because, for most elements, there appears to be a correlation with weight and total elemental concentration. But depending on the particular question, a hybrid approach may be more appropriate. To ensure that these approaches are possible for other scientists, our data, including kernel weight, are all available from www.ionomicshub.org through the data exchange portal.

### Conclusions

Here we have shown that single-seed-based ionomics is a robust and information-rich approach to identifying genetic and environmental determinants of the elemental profile of maize kernels. The limitations and potential artifacts are not large enough to prevent detection of statistically significant effects and represent a reasonable compromise to increase the scope and efficiency of investigation over absolute technical accuracy.

## Materials and Methods

### Biological Samples

The 25 NAM Founders were received from the U.S. Department of Agriculture Germplasm Resource Information Network (GRIN) at Ames, IA and grown at the Purdue Agronomy Center for Research and Education (West Lafayette, IN). The Founders of the maize Nested Association Mapping Panel were grown in a randomized block design, in 2010 (six blocks) and 2011 (five blocks). The soil type is a Udollic Ochraqualf silt loam. Plots were irrigated in late July of 2011 because of high temperatures and low rainfall. The fertilizer regimen consisted of 336 kg ha^−1^ potash (0-0-60) in the fall of both years and 123 kg ha^−1^ in 2010 and 2011 of anhydrous ammonia (81-0-0) with N-Serve® (DOW Agrosciences, Indianapolis, IN) prior to planting each year. To suppress weeds, the plots were treated at planting with 5.9 L ha^−1^ Harness Xtra® (Monsanto Company, St. Louis, MO), 0.84 kg ha^−1^ Princep® (Syngenta Crop Protection, Greensboro, NC), 0.57 kg ha^−1^ atrazine (Syngenta Crop Protection, Greensboro, NC), 1.75 L ha^−1^ Roundup® (Monsanto Company, St. Louis, MO), cultivated in mid-June, and subsequently hand-weeded through the growing season. Plots consisted of a single 3-m row with 76 cm between rows. Average stand density was 11 plants per row in 2010 and 15 plants per row in 2011. Lines were open pollinated and the seed harvested after maturity. Seed from the middle of the ear of one or two cobs per plot was removed from the cob by hand and analyzed using the ionomics pipeline.

For the heterogeneity within/between cobs experiment, the B73 accessions were received as gifts from Dr. Sherry Flint-Garcia (USDA ARS/Missouri University, Columbia, MO), Dr. Seth Murray (Texas A&M, College Station, TX), and Dr. Michael Muszynski (Iowa State, Ames, IA). Intact, hand-pollinated B73 ears were sampled from their 2009 nursery fields. Lines from the B73 x IL14H population were grown at the Illinois Crop Sciences Research and Education Center farm in 2007 using standard agronomic practices, with a single plot per line. Hand-pollinated ears were used to generate grain samples for this analysis. The soil properties of each site are listed in Table 5.

**Table 5:** Soil and Yield properties of growth locations.

Site Name	Site Location	PrimarySoil Type	Soil Classification	Average Estimated Maize Yield Mt ha^−1^
ISU Agronomy and Ag Engineering Research Farm	Boone IA	Clarion loam	Typic Hapludoll	11.5
OD Butler Jr Animal Science Complex and University Farm	Burleson TX	Boonville fine sandy loam	Chromic Vertic Albaqualf	2.9
Bradford Research and Extension Center	Columbia MO	Mexico silt loam	Vertic Epiaqualf	11.0
Purdue Agronomy Center for Research Education	West Lafayette IN	Toronto-Millbrook complex	Udollic Epiaqualf	8.6
Musgrave Research Farm	Poplar Ridge NY	Lima silt loam	Oxyaquic Hapludalf	7.5
Crop Sciences Research and Education Center	Urbana IL	Drummer silty loam	Typic Endoaquoll	11.0

### Determination of Elemental Concentration by ICP-MS Analysis

The analysis methods used are almost identical to those described for soybeans in Ziegler et al. [23]. As the precise methods of the pipeline are essential and integral to this manuscript, we have included identical or near-identical descriptions in this section. Identical passages are denoted by quotation marks.

#### Sample preparation and digestion

The seeds for the B73 x IL14H and B73 cob location experiments were weighed by hand. For the “Seeds were sorted into 48-well tissue culture plates, one seed per well. A weight for each individual seed was determined using a custom built weighing robot. The weighing robot holds six 48-well plates and maneuvers each well of the plates over a hole which opens onto a 3-place balance. After recording the weight, each seed was deposited using pressurized air into a 16×110 mm borosilicate glass test tube for digestion. The weighing robot can automatically weigh 288 seeds in approximately 1.5 hours with little user intervention.”

“Seeds were digested in 2.5 mL concentrated nitric acid (AR Select Grade, VWR) with internal standard added (20 ppb In, BDH Aristar Plus). Seeds were soaked at room temperature overnight, then heated to 105°C for two hours. After cooling, the samples were diluted to 10 mL using ultrapure 18.2 MΩ water (UPW) from a Milli-Q system (Millipore). Samples were stirred with a custom-built stirring rod assembly, which uses plastic stirring rods to stir 60 test tubes at a time. Between uses, the stirring rod assembly was soaked in a 10% HNO_3_ solution. A second dilution of 0.9 mL of the 1st dilution and 4.1 mL UPW was prepared in a second set of test tubes. After stirring, 1.2 mL of the second dilution was loaded into 96 well autosampler trays.”

#### ICP-MS Analysis

For the NAM parent experiments, elemental “concentrations of B, Na, Mg, Al, P, S, K, Ca, Mn, Fe, Co, Ni, Cu, Zn, As, Se, Rb, Mo, and Cd were measured using an Elan 6000 DRC-e mass spectrometer (Perkin-Elmer SCIEX) connected to a PFA microflow nebulizer (Elemental Scientific) and Apex HF desolvator (Elemental Scientific). Samples were introduced using a SC-FAST sample introduction system and SC4-DX autosampler (Elemental Scientific) that holds six 96-well trays (576 samples). “ The other experiments were run without the FAST sample introduction system and with a standard Apex desolvator (Elemental Scientific).

“All elements were measured with DRC collision mode off. Before each run, the lens voltage and nebulizer gas flow rate of the ICP-MS were optimized for maximum Indium signal intensity (>25,000 counts per second) while also maintaining low CeO+/Ce+ (<0.008) and Ba++/Ba+ (<0.1) ratios. This ensures a strong signal while also reducing the interferences caused by polyatomic and double-charged species. Before each run a calibration curve was obtained by analyzing six dilutions of a multi-element stock solution made from a mixture of single-element stock standards (Ultra Scientific).” In addition for the NAM parent experiment, “to correct for machine drift both during a single run and between runs, a control solution was run every tenth sample. The control solution is a bulk mixture of the remaining sample from the second dilution. Using bulked samples ensured that our controls were perfectly matrix matched and contained the same elemental concentrations as our samples, so that any drift due to the sample matrix would be reflected in drift in our controls. The same control mixture was used for every ICP-MS run in the project so that run-to-run variation could be corrected. A run of 576 samples took approximately 33 hours with no user intervention. The time required for cleaning of the instrument and sample tubes as well as the digestions and transfers necessary to set up the run limit the throughput to three 576 sample runs per week.”

#### Drift Correction and Analytical Outlier Removal

“Because our internal standard (IS) is added to our digesting acid, we are able to correct for losses due to differential sample evaporation, human error during the dilution process, and any sample introduction variability. So, if the final observed IS concentration is lower or higher than the starting IS concentration, all analyte concentrations are corrected equally for the percent difference between the observed IS concentration and the known starting IS concentration. IS correction is handled automatically by the PerkinElmer Elan 6000 software. Additionally, drift was corrected using the values of the controls run every tenth sample using a method similar to Haugen [24]. In short, drift was corrected by calculating the percentage of concentration change between two controls. This percentage change was assumed to have occurred linearly during the sequence of ten samples run between the two controls. So, for instance, the first sample run after the first control was corrected for 1/10^th^ of the drift seen between the two controls. Finally, because responses from the machine may be different between runs, we also corrected for drift between runs. This was performed by calculating a correction factor from the control concentrations in this run and a reference control used for all the runs. After drift correction, samples were corrected for the dilution factor and normalized to the seed weights.”

“While biological outliers are of great interest to our analysis, analytical outliers (e.g. from contamination, spurious isobaric and polyatomic interferences, or poor sample uptake) introduce noise and could lead to a higher rate of incorrectly chosen potential mutants. Outlier removal was implemented using the algorithm described in Davies and Gather [14]. To remove outliers while ensuring that we weren't removing biologically significant data points we removed data points on a per element basis from seeds whose reported elemental concentration was greater than a conservatively set 15 median absolute deviations from the median concentration of that element for that line.”

#### Computational Analysis

All calculations were performed using a combination of custom Perl and R scripts. The following R packages were used in the data analysis: reshape [25], ggplot. Standard Perl and R functions were used for drift correction, statistical analysis and creation of the figures. Scripts used in this analysis are available from http://www.ionomicshub.org, in the data exchange portal.

#### QTL analysis

To create the marker map, we pulled from the core set of 1100 NAM markers all the markers that were segregating in the B73 x IL14H population and removed highly correlated (r^2^>0.91) markers. We then computed a map using the Emap function in QTL Cartographer and verified co-linearity with the full map using all 1100 markers. We performed composite interval mapping (CIM) using QTL Cartographer version 1.17f [26], with CIM [27], [28] model 6, a walk speed of 2 cM, a window of 5 cM, using the forward and backward regression method. To determine threshold values, the permutation method was used [29] with 1000 permutations per element per population.
